# Analysis of medication errors by RCA method and implementation of reducing strategies to ‎improve patient safety in Hujjat Kuh-Kamari Hospital in Marand - 2017‎ 

**Published:** 2019-08

**Authors:** Tayyebeh Rezaei

**Affiliations:** ^*a*^Tabriz University of Medical Sciences, Tabriz, Iran.

## Abstract

**Background:**

Medical errors are one of the major challenges which threaten patient’s safety. Meanwhile, ‎medication errors are common types of medical errors that have attracted the attention of many ‎people. It is known as the eighth cause of death in USA. It has also caused many different ‎injuries, death, or increase in medical costs in other countries. Regarding the importance of ‎patient's safety and reduction of medical errors, this study was conducted to evaluate and ‎analyze of medication errors and implementation of strategies to reduce errors in Hujjat Kuh-‎Kamari Hospital in 2017.‎

**Methods:**

This research is an analytical and interventional study. All reported errors in 2017 were summed ‎up by personnel and patient safety staffs at monthly medical units and analyzed using the RCA ‎‎(Root Cause Analysis) by medical error analysis team and were ultimately analyzed in Ishikawa ‎diagram. Then, by identifying CPD (Caring Problem Delivery) and the SPD (System Problem ‎Delivery) factors, the main causes of these errors were evaluated from the moment of ‎prescribing and filing until giving the medication; then appropriate strategies were provided and ‎approved to reduce these errors by the Error Analysis Team. These strategies were then ‎provided to the treatment units. Finally, a reassessment of medication errors was performed ‎again in 2018 and compared with the results obtained in 2018 to determine the strategies ‎effectiveness.‎

**Results:**

The results of the study showed that the errors were in different forms, such as incorrect dose, ‎incorrect registration of the drug, lack of registration of the drug, incorrect entry at list, failure ‎to implement it, and wrong implementation of medication order. According to the analysis of ‎the errors, the causes of the occurrence of these errors were the cases such as incorrect checking ‎of the orders (20%), the problem with readability of the order (15.5%), the similarity of the ‎medication in terms of the name, form and appearance (14%) the incomplete written name of ‎medication by the doctor (7.8%) the incomplete written name of medication by the nurse ‎‎(8.7%), and other errors. Ultimately, solutions were provided to the units as follows:‎

• Checking the orders again by the next person in the next shift and matching it with the ‎orders in patient's list.‎

• Matching the medications in patient's list and records by the secretary of the unit at the ‎time of registration in HIS system.‎

• Sending letter to doctors to write the medication orders better and in a readable way.‎

• Separating similar medications and identifying the risky and similar medications with ‎yellow and red labels. ‎

These errors were assessed in 2018 again, and all errors were reduced by an average of 8.35%. ‎However, the errors related to doctors' handwriting were increased up to 0.4%.‎

**Conclusions:**

Considering the importance of patient safety and receiving safe services in hospitals, it is ‎important to identify errors and its causes, examine the strengths and weaknesses of reporting ‎errors, share errors and find ways to reduce or eliminate these errors for improving patient ‎safety in the hospital. Therefore, it is necessary to create organizational culture and patient ‎safety culture in order to feel responsible for the system and patients in the medical staff. In this ‎regard, encouraging staff to report errors, contributing them to the analyze errors, using staff ‎comments, considering the management's responsibility about patient safety, reporting errors, ‎and encouraging staff who are pioneer in patient safety advancement can be very helpful.‎

**Keywords:**

Patient safety, Medication errors, Root cause analysis

